# Classical-to-quantum transition behavior between two oscillators separated in space under the action of optomechanical interaction

**DOI:** 10.1038/s41598-017-02779-w

**Published:** 2017-05-31

**Authors:** Cheng-Hua Bai, Dong-Yang Wang, Hong-Fu Wang, Ai-Dong Zhu, Shou Zhang

**Affiliations:** Department of Physics, College of Science, Yanbian University, Yanji, Jilin 133002 China

## Abstract

We propose a scheme to show that the system consisting of two macroscopic oscillators separated in space which are coupled through Coulomb interaction displays the classical-to-quantum transition behavior under the action of optomechanical coupling interaction. Once the optomechanical coupling interaction disappears, the entanglement between the two separated oscillators disappears accordingly and the system will return to classical world even though there exists sufficiently strong Coulomb coupling between the oscillators. In addition, resorting to the squeezing of the cavity field generated by an optical parametric amplifier inside the cavity, we discuss the effect of squeezed light driving on this classical-to-quantum transition behavior instead of injecting the squeezed field directly. The results of numerical simulation show that the present scheme is feasible and practical and has stronger robustness against the environment temperature compared with previous schemes in current experimentally feasible regimes. The scheme might possibly help us to further clarify and grasp the classical-quantum boundary.

## Introduction

Quantum entanglement^1, 2^, a most remarkable feature and a cornerstone of quantum physics, plays a significant role in the foundation of quantum theory and also has potential applications in quantum technology, such as quantum information science^3^ and quantum metrology^4^. So far, one has had a fairly good understanding of how to generate entanglement among microscopic entities and entanglement has been successfully prepared and manipulated in variously microscopic systems theoretically and experimentally, such as atoms^5–9^, photons^10–12^, ions^13–15^. Bose-Einstein condensates^16^, and so on. However, it is not yet completely clear that to what degree quantum mechanics is suitable for mesoscopic and macroscopic systems and the generation of quantum entanglement of mesoscopic or macroscopic bodies in mechanical motion is generally bounded by the thermal fluctuation exerted by their environments. Recently, there has been considerable interest in investigating entanglement in mesoscopic and even macroscopic systems^17–28^. This is due to the fact that such entanglement might provide explicit evidence for quantum phenomena^29^ and even might possibly help us to clarify the classical-to-quantum transition, as well as the boundary between classical and quantum worlds^30^. Since mechanical oscillators resemble a prototype of classical systems, they are beginning to be important candidates for the investigation of quantum features at mesoscopic and macroscopic scales. Additionally, with the rapid progress of practical technologies in cavity optomechanics, the mechanical oscillators can be cooled down close to the quantum ground state^31–33^. Thus they provide a nature platform to explore non-classical effects in macroscopic systems^34, 35^.

In recent years, based on the optomechanical systems, some schemes have been brought forward to generate entanglement between macroscopic oscillators from many different angles of view: such as entangling two oscillators in a ring cavity^17, 18^, entangling two distantly separated oscillators by utilizing the entangled light fields^19^, entangling two oscillators via a double-cavity set-up by driving squeezing optical fields^20^, entangling a Fabry-Pérot cavity’s two moving mirrors by driving an intense classical laser field^21^, entangling two dielectric membranes suspended inside a Fabry-Pérot cavity^22^, entangling two macroscopic mechanical resonators induced by the radiation pressure of a single photon in a two-cavity optomechanical system^23^, generating steady-state entanglement of remote mechanical oscillators in unidirectionally coupled cavities by the cascaded cavity coupling^24^, realizing entanglement of spatially separated mechanical modes via applying a bichromatic pump leads to time-dependent optical springs that can tune couplings between nondegenerate mechanical modes into the type of parametric amplifier resonance^25^, generating entanglement between two mechanical resonators with different frequencies either dynamically or in the steady state^26^, and entangling two movable mirrors in an optomechanical cavity in which a Kerr-down-conversion crystal consisting of a Kerr nonlinear medium and an optical parametric amplifier (OPA) is placed^27^. In these schemes, we can roughly classify these mechanical entanglement state-generation schemes into two categories, according to the entanglement is created by purely optomechanical means^17, 22–26^, or is adjusted by the squeezed field^18–21, 27^. Huang and Agarwal proposed a scheme to entangle two separated mechanical oscillators by injecting broad band squeezed vacuum light and laser light into the ring cavity^18^. This scheme showed that the entanglement can be modulated via the squeezing parameter of the input light. In the case of no injection of the squeezed vacuum light, which means that the squeezed vacuum light is replaced by the ordinary vacuum light, there is always no entanglement between the separated oscillators. However, once the incident vacuum light is squeezed, the entanglement exists. Pinard *et al*. also proposed a scheme to generate a stationary entangled state of two movable mirrors if and only if the incident fields are squeezed^20^. In ref. 27 even though the entanglement between two mechanical oscillators in an optomechanical cavity can be generated when the injected field is not squeezed, the region of entanglement is discrete and very narrow, so which inevitably brings difficulties to achieve the entanglement in experiment. However, when the injected field is adjusted to the squeezed field, the region of entanglement is continuous and greatly enlarged. In essence, the OPA inside the optomechanical cavity can produce various novel effects including improvement of the cooling of the micromechanical mirror^36^, affection of the normal-mode splitting behavior of the coupled movable mirror and the cavity field^37^, achievement of strong mechanical squeezing^38^, and enhancement of the precision of optomechanical position detection^39^. The nonlinear interaction processes between light and OPA have been demonstrated as important sources of squeezed state of the radiation field^40, 41^. In 2016, Agarwal and Huang have had the OPA placed inside the optomechanical cavity so that the squeezing cavity field is generated inside the cavity^38^. Via driving the system by the red-detuned laser in the resolved sideband limit makes the optomechanical interaction between the movable mirror and the cavity field like a beam-splitter interaction, the state of squeezed photons transfers to phonons with almost 100% efficiency, the strong mechanical squeezing is thus achieved.

Recently, the hybrid quantum system consisting of two coupled resonators has been investigated^42, 43^. Here we propose a scheme to show that the system consisting of two macroscopic oscillators separated in space which are coupled through Coulomb interaction displays the classical-to-quantum transition behavior under the auxiliary of the optomechanical coupling interaction. Our investigation indicates that once the optomechanical coupling interaction disappears, the entanglement between the two separated oscillators disappears accordingly and the system will return to classical world even though there exists sufficiently strong Coulomb coupling between the oscillators. Resorting to the squeezing of the cavity field generated by an OPA inside the cavity, we discuss the effect of squeezed light driving on this classical-to-quantum transition behavior instead of injecting the squeezed field directly. In current experimentally feasible regimes, the results of numerical simulation show that the present scheme is feasible and practical and has stronger robustness against the environment temperature compared with previous schemes. It is promising that the scheme might possibly help us to further clarify and grasp the classical-quantum boundary.

The paper is organized as follows. In Sec. 2, we describe the model and present the quantum Langevin equations. In Sec. 3, we present the definition of the covariance matrix and transform the quantum Langevin equations into a equivalent differential equation of the covariance matrix. In Sec. 4, we show that the system consisting of two macroscopic oscillators separated in space which are coupled through Coulomb interaction displays the classical-to-quantum transition behavior under the auxiliary of optomechanical coupling interaction and discuss the effect of squeezed light driving generated by the OPA inside the cavity on this classical-to-quantum transition behavior in current experimentally feasible steady regime. Finally we make a conclusion to summarize our results in Sec. 5.

## Results

### Model and equations of motion

**Figure 1:**
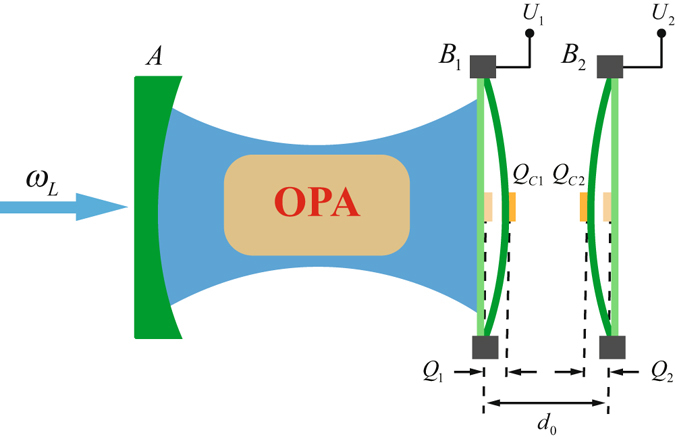
Schematic diagram of the system. The cavity optomechanical system consists of a fixed mirror *A* and a mechanical oscillator *B*_1_ which is coupled to the other mechanical oscillator *B*_2_ under the action of the Coulomb interaction. An OPA is placed inside the cavity, and the pump of the OPA is not shown. The cavity is driven by the driving field *E* with frequency *ω*_*L*_. The electrode carrying charge *Q*_*C*1_ (*Q*_*C*2_) on *B*_1_ (*B*_2_) is charged by the bias gate voltage *U*_1_ (*U*_2_). *d*_0_ is equilibrium separation of *B*_1_ and *B*_2_. *Q*_1_ and *Q*_2_ are the small deviation of *B*_1_ and *B*_2_ from their equilibrium positions, respectively, due to the radiation pressure interaction and Coulomb interaction.

The system considered consists of a Fabry-Pérot cavity containing one fixed partially transmitting mirror *A* and one movable totally reflecting mirror *B*_1_ in contact with a thermal bath in equilibrium at temperature *T*, a charged oscillator *B*_2_, and an OPA which is embedded into the cavity, as schematically shown in Fig. 1. The movable mirror *B*_1_ can move along the cavity axis and is treated as a mechanical harmonic oscillator with effective mass *m*, frequency *ω*_1_, and energy decay rate ${\gamma }_{{m}_{1}}$ and is charged by the bias gate voltage *U*_1_. The cavity mode couples to the mechanical oscillator *B*_1_ via radiation pressure caused by the intracavity photons exerting on the movable mirror, while *B*_1_ and *B*_2_ are coupled by the Coulomb force^44–47^. The cavity is coherently driven by an external laser with frequency *ω*_*L*_ and amplitude *E* from left side. The Hamiltonian of the system is given by

1 $$\begin{array}{rcl}H & = & \hslash {\omega }_{c}{c}^{\dagger }c+\frac{\hslash {\omega }_{m1}}{2}({p}_{1}^{2}+{q}_{1}^{2})+\frac{\hslash {\omega }_{m2}}{2}({p}_{2}^{2}+{q}_{2}^{2})+i\hslash E({c}^{\dagger }{e}^{-i{\omega }_{L}t}-c{e}^{i{\omega }_{L}t})\\  &  & -\,\hslash {G}_{0}{c}^{\dagger }c{q}_{1}+i\hslash {C}_{g}({e}^{i\theta }{c}^{\dagger 2}{e}^{-2i{\omega }_{L}t}-{e}^{-i\theta }{c}^{2}{e}^{2i{\omega }_{L}t})+\frac{-{k}_{e}{Q}_{C1}{Q}_{C2}}{|{d}_{0}+{Q}_{1}-{Q}_{2}|},\end{array}$$

where the first term is the free Hamiltonian for the cavity field with resonance frequency *ω*_*c*_ and annihilation (creation) operator *c* (*c* ^†^). The second and third terms describe the vibration of the mechanical oscillators *B*_1_ and *B*_2_, respectively, and the dimensionless position operator ${q}_{j}=\frac{1}{\sqrt{2}}\frac{{Q}_{j}}{{x}_{zp}}$ and momentum operator ${p}_{j}=\frac{1}{\sqrt{2}}\frac{{P}_{j}}{{p}_{zp}}$ satisfy the commutation relation [*q*_*j*_, *p*_*j*_] = *i* (*j* = 1, 2), where *x*_*zp*_ and *p*_*zp*_ are, respectively, the stand deviation of the zero-point motion and zero-point momentum of the oscillator. The fourth term is the pumping interaction between the cavity field and external deriving laser with $E=\sqrt{2\kappa P/\hslash {\omega }_{L}}$, where *P* is the power of the driving laser and *κ* is the cavity decay rate. The fifth term describes the optomechanical interaction between the cavity field and the mechanical oscillator *B*_1_ with the optomechanical coupling strength ${G}_{0}=({\omega }_{c}/L)\sqrt{\hslash /m{\omega }_{m1}}$, where *L* is the separation between the mirror *A* and oscillator *B*_1_ in the absence of radiation pressure and Coulomb interactions. The sixth term represents the coupling between the OPA and the cavity field, where *C*_*g*_ is the nonlinear gain of the OPA and *θ* is the phase of the pump driving the OPA, i.e., the phase shift between the signal (idler) mode and the pump mode. The last term represents the Coulomb interaction of the two charged mechanical oscillators *B*_1_ and *B*_2_. *k*_*e*_ denotes the electrostatic constant. *Q*_*Cj*_ = *C*_*j*_ *U*_*j*_ is the charge carried by the electrode on oscillator *B*_*j*_, where *C*_*j*_ is the capacitance of the bias gate on *B*_*j*_. *d*_0_ is the equilibrium separation between *B*_1_ and *B*_2_ in the absence of optomechanical and Coulomb interactions and *Q*_*j*_ represents the small deviation of *B*_*j*_ from its equilibrium position due to the optomechanical and Coulomb interactions.

Since the mechanical deviation *Q*_*j*_ is comparatively small compared to the equilibrium separation *d*_0_, i.e., *Q*_*j*_ ≪ *d*_0_, the term of Coulomb interaction can be expanded to second-order of (*Q*_1_ − *Q*_2_)/*d*_0_ as follows

2 $${H}_{{\rm{C}}{\rm{I}}}=\frac{-{k}_{e}{C}_{1}{U}_{1}{C}_{2}{U}_{2}}{|{d}_{0}+{Q}_{1}-{Q}_{2}|}=\frac{-{k}_{e}{C}_{1}{U}_{1}{C}_{2}{U}_{2}}{{d}_{0}}[1-\frac{{Q}_{1}-{Q}_{2}}{{d}_{0}}+{(\frac{{Q}_{1}-{Q}_{2}}{{d}_{0}})}^{2}],$$

where the linear term can be neglected via redefining the equilibrium positions of mechanical oscillators and the quadratic term includes a renormalization of the mechanical frequencies for both *B*_1_ and *B*_2_. Through further discarding the constant term, the Coulomb interaction can be reduced to the simpler form

3 $${H}_{{\rm{CI}}}=\hslash \lambda {q}_{1}{q}_{2},$$

where $\lambda =2{k}_{e}{C}_{1}{U}_{1}{C}_{2}{U}_{2}/m{\omega }_{m}{d}_{0}^{3}$ ^44, 46–48^. In the interaction picture with respect to $\hslash {\omega }_{L}{c}^{\dagger }c$, the system Hamiltonian can be rewritten as

4 $$\begin{array}{rcl}H & = & \hslash ({\omega }_{c}-{\omega }_{L}){c}^{\dagger }c+\frac{\hslash {\omega }_{m1}}{2}({p}_{1}^{2}+{q}_{1}^{2})+\frac{\hslash {\omega }_{m2}}{2}({p}_{2}^{2}+{q}_{2}^{2})+\hslash \lambda {q}_{1}{q}_{2}\\  &  & +\,i\hslash E({c}^{\dagger }-c)-\hslash {G}_{0}{c}^{\dagger }c{q}_{1}+i\hslash {C}_{g}({e}^{i\theta }{c}^{\dagger 2}-{e}^{-i\theta }{c}^{2}).\end{array}$$

A proper analysis of the system must consider the photon losses from the cavity and the Brownian noise from the environment. This can be accomplished via the dynamics of the system governed by Eq. (4) using quantum Langevin equation

5 $$\begin{array}{rcl}{\dot{q}}_{1} & = & {\omega }_{m1}\,{p}_{1},\\ {\dot{p}}_{1} & = & -\,{\omega }_{m1}{q}_{1}-{\gamma }_{m1}\,{p}_{1}+{G}_{0}{c}^{\dagger }c-\lambda {q}_{2}+{\xi }_{1},\\ {\dot{q}}_{2} & = & {\omega }_{m2}\,{p}_{2},\\ {\dot{p}}_{2} & = & -\,{\omega }_{m2}{q}_{2}-{\gamma }_{m2}\,{p}_{2}-\lambda {q}_{1}+{\xi }_{2},\\ \dot{c} & = & -\,[\kappa +i({\omega }_{c}-{\omega }_{L})]c+i{G}_{0}c{q}_{1}+E+2{C}_{g}{e}^{i\theta }{c}^{\dagger }+\sqrt{2\kappa }{c}_{in},\end{array}$$

where *γ*_*m*2_ is the damping rate for the oscillator *B*_2_. *c*_*in*_ is the input vacuum noise operator with zero mean value and nonzero correlation function $\langle {c}_{in}(t){c}_{in}^{\dagger }(t^{\prime} )\rangle =\delta (t-t^{\prime} )$ ^19, 49, 50^. The quantum Brownian noise *ξ*_1_ *(ξ*_2_) arises from the coupling between *B*_1_ (*B*_2_) and its environment with zero mean value and correlation function^51^

6 $$\langle {\xi }_{j}(t){\xi }_{j}(t^{\prime} )\rangle =\frac{{\gamma }_{mj}}{{\omega }_{mj}}\int \frac{\omega }{2\pi }{e}^{-i\omega (t-t^{\prime} )}[\coth (\frac{\hslash \omega }{2{k}_{B}T})+1]d\omega ,$$

where *k*_*B*_ is the Boltzmann constant and *T* is the temperature of the environment in contact with the oscillators. However, quantum effects are revealed just for the oscillators with a large quality factor, i.e., *Q* ≫ 1. In this limit, Eq. (6) can be further simplified to delta-correlated^51^

7 $$\langle {\xi }_{j}(t){\xi }_{j}(t^{\prime} )+{\xi }_{j}(t^{\prime} ){\xi }_{j}(t)\rangle /2\simeq {\gamma }_{mj}(2\bar{n}+1)\delta (t-t^{\prime} ),$$

where $\bar{n}={(\exp \{\hslash {\omega }_{mj}/{k}_{B}T\}-1)}^{-1}$ is the mean thermal excitation number. In the following we present the definition of the covariance matrix and transform the Langevin equations into a equivalent differential equation of the covariance matrix.

### Equation of motion for covariance matrix

The stability of the steady state of the system is determined by a linearized analysis for small perturbation around the steady state^41^. We now first linearize the dynamics of the system. The nonlinear quantum Langevin equations can be linearized via rewriting each Hersenberg operator as its steady state mean-value plus an additional fluctuation operator with zero-mean value, i.e., *q*_*j*_ = *q*_*js*_ + *δq*_*j*_, *p*_*j*_ = *p*_*js*_ + *δp*_*j*_, and *c* = *c*_*j*_ + *δc* ^52^. After inserting these expressions into the Langevin equations of Eq. (5), we can obtain a set of nonlinear algebraic equations for the steady state values and a set of quantum Langevin equations for the fluctuation operators^53^. Through setting all the time derivatives in algebra equations for the steady state value to zero, the steady state mean values of system are given by

8 $$\begin{array}{rcl}{p}_{1s} & = & 0,\\ {q}_{1s} & = & \frac{{G}_{0}{|{c}_{s}|}^{2}}{{\omega }_{m1}-\frac{{\lambda }^{2}}{{\omega }_{m2}}},\\ {p}_{2s} & = & 0,\\ {q}_{2s} & = & \frac{-\lambda }{{\omega }_{m2}}{q}_{1s},\\ {c}_{s} & = & \frac{\kappa -i{\rm{\Delta }}+2{C}_{g}{e}^{i\theta }}{{\kappa }^{2}+{{\rm{\Delta }}}^{2}-4{C}_{g}^{2}}E,\end{array}$$

where Δ = *ω*_*c*_ − *ω*_*L*_ − *G*_0_ *q*_1*s*_ is the effective cavity detuning from the frequency of the input laser in the presence of the radiation pressure. The modification of the detuning by the *G*_0_ *q*_1*s*_ term depends on the mechanical motion. The *q*_1*s*_ represents the new equilibrium position of the oscillator *B*_1_ relative to that in the absence of the optomechanical and Coulomb interactions and *c*_*s*_ denotes the steady state amplitude of the cavity field.

In order to analyze the oscillator-oscillator steady state entanglement, we need to find out the fluctuations in the oscillators’ amplitudes. So we are interested in the dynamics of small fluctuations around the steady state of the system. For generating the entanglement, generally, the cavity is intensively driven with a very large input power *P*, which means that at the steady state, the intracavity field has a large amplitude, i.e., |*c*_*s*_| ≫ 1. In this strong driving limit, we can ignore some small quantities and get the linearized Langevin equations

9 $$\begin{array}{rcl}\delta {\dot{q}}_{1} & = & {\omega }_{m1}\delta {p}_{1},\\ \delta {\dot{p}}_{1} & = & -\,{\omega }_{m1}\delta {q}_{1}-{\gamma }_{m1}\delta {p}_{1}-\lambda \delta {q}_{2}+{G}_{0}({c}_{s}^{\ast }\delta c+{c}_{s}\delta {c}^{\dagger })+{\xi }_{1},\\ \delta {\dot{q}}_{2} & = & {\omega }_{m2}\delta {p}_{2},\\ \delta {\dot{p}}_{2} & = & -\,\lambda \delta {q}_{1}-{\omega }_{m2}\delta {q}_{2}-{\gamma }_{m2}\delta {p}_{2}+{\xi }_{2},\\ \delta \dot{c} & = & -\,(\kappa +i{\rm{\Delta }})\delta c+i{G}_{0}{c}_{s}\delta {q}_{1}+2{C}_{g}{e}^{i\theta }\delta {c}^{\dagger }+\sqrt{2\kappa }{c}_{in}.\end{array}$$

If we choose the phase reference of the cavity field so that *c*_*s*_ is real via adjusting the phase of the driving laser to ${\rm{arc}}\,\tan [\frac{{\rm{\Delta }}-2{C}_{g}\,\sin \,\theta }{\kappa +2{C}_{g}\,\cos \,\theta }]$ and introduce the amplitude and phase fluctuations of the cavity field as $\delta X=(\delta c+\delta {c}^{\dagger })/\sqrt{2}$ and $\delta Y=(\delta c-\delta {c}^{\dagger })/\sqrt{2}i$, and the position and momentum fluctuations of the thermal noise as ${X}^{in}=({c}_{in}+{c}_{in}^{\dagger })/\sqrt{2}$ and ${Y}^{in}=({c}_{in}-{c}_{in}^{\dagger })/\sqrt{2}i$, Eq. (9) can be written as the matrix form

10 $$\dot{f}(t)=Mf(t)+\eta (t),$$

where *f*(*t*) is the column vector of the fluctuations and *η*(*t*) is the column vector of the noise sources. Their transposes are

11 $$f{(t)}^{T}=(\delta {q}_{1},\delta {p}_{1},\delta {q}_{2},\delta {p}_{2},\delta X,\delta Y),\eta {(t)}^{T}=(0,{\xi }_{1},0,{\xi }_{2},\sqrt{2\kappa }{X}^{in},\sqrt{2\kappa }{Y}^{in});$$

and the matrix *M* is given by

12 $$M=[\begin{array}{cccccc}0 & {\omega }_{m1} & 0 & 0 & 0 & 0\\ -{\omega }_{m1} & -{\gamma }_{m1} & -\lambda  & 0 & {G}_{m} & 0\\ 0 & 0 & 0 & {\omega }_{m2} & 0 & 0\\ -\lambda  & 0 & -{\omega }_{m2} & -{\gamma }_{m2} & 0 & 0\\ 0 & 0 & 0 & 0 & 2{C}_{g}\,\cos \,\theta -\kappa  & 2{C}_{g}\,\sin \,\theta +{\rm{\Delta }}\\ {G}_{m} & 0 & 0 & 0 & 2{C}_{g}\,\sin \,\theta -{\rm{\Delta }} & -(2{C}_{g}\,\cos \,\theta +\kappa )\end{array}],$$

where ${G}_{m}=\sqrt{2}{G}_{0}{c}_{s}$ is the effective optomechanical coupling. Remarkably, the quantum fluctuations of the field and the oscillator are now coupled by the much large effective coupling.

The solutions to Eq. (10) are stable only if all the eigenvalues of the matrix *M* have negative real parts. The stability conditions can be derived by applying the Routh-Hurwitz criterion^54, 55^, yielding the constrain conditions on the system parameters. Due to their expressions are considerable tedious, we don’t report them here. However, we will satisfy the stability conditions of the system in the following analysis.

The solution of the first-order linear inhomogeneous differential Eq. (10) can be solved as following form

13 $$f(t)=u(t)f(0)+{\int }_{0}^{t}u(\tau )\eta (t-\tau )d\tau ,$$

where the matrix *u*(*t*) = exp(*Mt*) and the initial condition *u*(0) = *I* (*I* is the identity matrix).

An important type of continuous variable quantum states is the Gaussian states, which play a significant role in the foundation of quantum theory and also have potential applications in their relevant experiment^56^. The linearized effective Hamiltonian which corresponds to the linearized Langevin Eq. (9) ensures that when the system is stable, it always reaches a Gaussian state whose information-related properties, such as entanglement and entropy, can be completely described by the symmetric 6 × 6 covariance matrix *V* ^56, 57^ with components defined as

14 $${V}_{i,j}=\langle {f}_{i}{f}_{j}+{f}_{j}{f}_{i}\rangle /2.$$

From Eqs (10) and (14), we can derive a linear differential equation for the covariance matrix

15 $$\dot{V}=MV+V{M}^{T}+D,$$

where *D* is a diffusion matrix whose components are associated with the noise correlation function Eq. (7)

16 $${D}_{i,j}\delta (t-t^{\prime} )=\langle {\eta }_{i}(t){\eta }_{j}(t^{\prime} )+{\eta }_{j}(t^{\prime} ){\eta }_{i}(t)\rangle /2.$$

It is easy to obtain that *D* is diagonal $D={\rm{diag}}[0,{\gamma }_{m1}(2\bar{n}+1),0,{\gamma }_{m2}(2\bar{n}+1),\kappa ,\kappa ]$. From the point of view of describing the dynamics of the system Gaussian states, Eq. (15) is equivalent to the quantum Langevin equations Eq. (9) but is more convenient for studying entanglement evolution.

The reduced 4 × 4 covariance matrix $\mathop{V}\limits^{ \sim }$ for the mechanical oscillators *B*_1_ and *B*_2_ of interest here can be extracted from the full 6 × 6 covariance matrix *V*. If the reduced covariance matrix $\mathop{V}\limits^{ \sim }$ is written as the block form

17 $$\mathop{V}\limits^{ \sim }=[\begin{array}{cc}{{\rm{\Phi }}}_{1} & {{\rm{\Phi }}}_{3}\\ {{\rm{\Phi }}}_{3}^{T} & {{\rm{\Phi }}}_{2}\end{array}],$$

where Φ_*k*_(*k* = 1, 2, 3) are 2 × 2 block matrices, then the entanglement of the two separated mechanical oscillators *B*_1_ and *B*_2_ quantified by the logarithmic negativity can be readily calculated^58–60^

18 $${E}_{N}=\,{\rm{\max }}\,[0,-\mathrm{ln}(2\rho )],$$

where $\rho \equiv {2}^{-1/2}{\{{\rm{\Sigma }}(V)-{[{\rm{\Sigma }}{(V)}^{2}-4{\rm{\det }}V]}^{1/2}\}}^{1/2}$, with ${\rm{\Sigma }}(V)\equiv {\rm{\det }}\,{{\rm{\Phi }}}_{1}+{\rm{\det }}\,{{\rm{\Phi }}}_{2}-2{\rm{\det }}\,{{\rm{\Phi }}}_{3}$. Therefore, a Gaussian state is entangled if and only if *ρ* < 1/2, which is equivalent to Simon’s necessary and sufficient entanglement nonpositive partial transpose criterion for Gaussian states^61^.

#### The classical-to-quantum transition behavior between two oscillators separated in space in the steady regime

In this section, we will show that the system consisting of two oscillators separated in space which are coupled through Coulomb interaction displays the classical-to-quantum transition behavior under the action of optomechanical coupling interaction and discuss the effect of squeezed light driving generated by the OPA inside the cavity on this classical-to-quantum transition behavior via numerically evaluating the logarithmic negativity *E*_*N*_ in current experimentally feasible steady regime. Here, we assume that all the parameters of the two mechanical oscillators to be the same, i.e., *ω*_*m*1_ = *ω*_*m*2_ = *ω*_*m*_, *γ*_*m*1_ = *γ*_*m*2_ = *γ*_*m*_. We choose the parameters in our numerical calculations are based on the experiment conditions^62, 63^: *ω*_*m*_ = 200*π* MHz, *γ*_*m*_ = 200*π* Hz, *κ* = 88.1 MHz, *m* = 5 ng, *L* = 1 mm, and the wavelength of driving laser *λ*_0_ = 810 nm.

**Figure 2:**
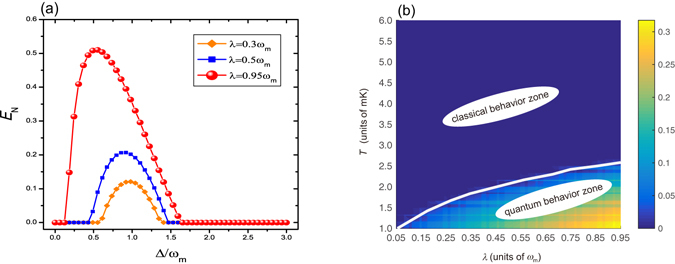
(**a**) Plot of the logarithmic negativity *E*_*N*_ as a function of the normalized detuning Δ/*ω*_*m*_ for three different values of the Coulomb coupling strength λ = 0.3 *ω*_*m*_ (orange diamond line), λ = 0.5 *ω*_*m*_ (blue square line), and λ = 0.95 *ω*_*m*_ (red sphere line) in the absence of the OPA (*C*_*g*_ = 0). Here the temperature of environment and driving power are set to *T* = 4 mK and *P* = 50 mW, respectively. (**b**) The logarithmic negativity *E*_*N*_ versus the environment temperature *T* and the Coulomb coupling strength *λ* in the absence of the optomechanical coupling between the optical field and the oscillator *B*_1_, i.e., *G*_0_ = 0. The rest parameters are chosen as *ω*_*m*_ = 200 *π*MHz and *γ*_*m*_ = 200 *π*Hz.

First, we illustrate the effect of the Coulomb interaction on the entanglement between the two separated mechanical oscillators in the absence of the OPA (*C*_*g*_ = 0), i,e., ordinary light driving. The logarithmic negativity *E*_*N*_ as a function of the normalized detuning Δ/*ω*_*m*_ for three different values of the Coulomb coupling strength *λ* = 0.3 *ω*_*m*_ (orange diamond line), *λ* = 0.5 *ω*_*m*_ (blue square line), and λ = 0.95 *ω*_*m*_ (red sphere line) at temperature *T* = 4 mK and driving power *P* = 50 mW in the absence of the OPA is shown in Fig. 2(a). As illustrated in previous section, as long as the logarithmic negativity *E*_*N*_ > 0, the entanglement exists between the two oscillators, meaning that there is a quantum correlation between them, even though they are separated in space. From Fig. 2(a), one can clearly see that the larger the coupling parameter *λ* is, the stronger the oscillators entangle and the broader the range of the entanglement is. The numerical result shows that if there is no the Coulomb coupling, it is not possible to entangle the two oscillators which are separated in space. Now, we consider the feasibility of the choice of numerical value of the coupling strength *λ* in experiment. If we apply the reported experimental parameters, i.e., the capacitance of the bias gate on *B*_1_ and *B*_2_ *C*_1_ = *C*_2_ = 2.4 nF, the equilibrium distance between *B*_1_ and *B*_2_ without optomechanical and Coulomb interactions d_0_ = 160 *μ*m^47^, and adjust the bias gate voltage *U*_1_ = *U*_2_ = 200 V, in this case, *λ* ≃ 0.33 *ω*_*m*_. Compared with the numerical value needed in our scheme, it has the same order of magnitude, so the choice of the numerical value of *λ* is feasible in experiment.

Subsequently, we consider the role of the optomechanical coupling between the optical field and the oscillator *B*_1_ plays in the generating entanglement between two separated oscillators in space. For this purpose, we make a density plot of *E*_*N*_ versus the environment temperature *T* and the Coulomb coupling strength *λ* in the absence of the optomechanical coupling, i.e., *G*_0_ = 0, in Fig. 2(b). As shown in Fig. 2(b), it can be clearly seen that in the case of absence of optomechanical coupling, if we decrease the environment temperature *T* to the range of quite low and the Coulomb coupling strength *λ* is quite strong, the system consisting of two separated oscillators in space will display the quantum entanglement behavior. But with the increase of temperature, the quantum entanglement behavior will disappear and the system returns to classical world, even though there exists the sufficiently strong Coulomb coupling between the two oscillators. This is the reason why we can not observe the quantum phenomena in macroscopic world generally. The higher the environment temperature is, the stronger the thermal noise is. Then the entanglement between the two separated oscillators in space is submerged by the strong thermal noise. So the generation of quantum entanglement of mesoscopic or macroscopic bodies in mechanical motion is generally bounded by the thermal noise exerted by their environments. However, under the auxiliary of the optomechanical coupling, the two separated oscillators in space in the classical behavior zone can display the quantum entanglement behavior. From Fig. 2, it can be clearly seen that once the optomechanical coupling between the optical field and oscillator *B*_1_ disappears, the entanglement between the two separated oscillators in space will disappear accordingly and the system returns to classical world. This is due to the optomechanical coupling between the optical field and oscillator cools down the oscillator and sufficiently suppresses the detrimental effect of the thermal noise. Hence, the optomechanical coupling between the optical mode and the mechanical mode provides a perfect platform for the study of this classical-to-quantum transition behavior.

In the previous schemes, ref. 18 proposed a method to entangle two separated oscillators by injecting squeezed vacuum light and laser light into the ring cavity. The entanglement between the oscillators can be modulated via the squeezing parameter of the input light. When the squeezed vacuum light is replaced by an ordinary vacuum light, i.e., the squeezing parameter of the input light is 0, there is no entanglement between the oscillators. However, on squeezing the injected vacuum light, the entanglement between the oscillators is emerged. When the squeezing parameter of the input light *r* ∈ (0, 1), the entanglement becomes more and more stronger with the increase of *r*, while for *r* ∈ (1, 2), the entanglement becomes more and more weaker with the increase of *r*. So in this scheme the entanglement between the separated oscillators is affected by the driven light is squeezed or not to a large extent. Ref. 27 also proposed to coherently control the entanglement between two movable mirrors via placing the Kerr-down-conversion crystal consisting of Kerr nonlinear medium and OPA inside an optomechanical cavity. By the aid of the input squeezed vacuum field, the Kerr nonlinear medium can lead to stronger entanglement between the two movable mirrors and extend to wider entanglement region. Whereas the effect of the OPA on entanglement is completely opposite, it leads weaker entanglement and narrower entanglement region. Likewise, the entanglement between two separated movable mirrors is remarkably affected by the input squeezed field. The above two schemes have the common point that they all resort to the external squeezed vacuum filed and are dependent in a important way on the driving of squeezed light. So a natural curiosity and question are that whether the driving of squeezed light will affect this classical-to-quantum transition behavior of the system consisting of two macroscopic oscillators separated in space or not and how to affect. As we all know, the nonlinear interaction processes between light and OPA have been considered as important sources of squeezed state of the radiation field^40, 41^. Fortunately, in 2016, Agarwal and Huang investigated the dependence of the amount of squeezing of the intracavity photons on the parametric gain and driving phase of an OPA^38^. Next, resorting to the squeezing of the cavity field generated by an OPA inside the cavity, we discuss the effect of squeezed light driving on this classical-to-quantum transition behavior instead of injecting the squeezed field directly.

**Figure 3:**
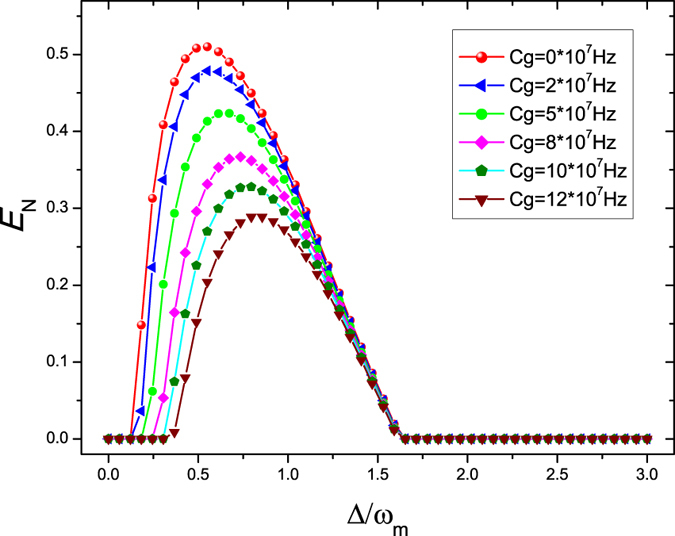
Plot of the logarithmic negativity *E*_*N*_ as a function of the normalized detuning Δ/*ω*_*m*_ for six different values of *C*_*g*_ = 0 (red sphere line), *C*_*g*_ = 2 × 10^7^ Hz (blue triangle line), *C*_*g*_ = 5 × 10^7^ Hz (green circle line), *C*_*g*_ = 8 × 10^7^ Hz (magenta diamond line), *C*_*g*_ = 10 × 10^7^ Hz (olive pentagon line), and *C*_*g*_ = 12×10^7^ H^*z*^ (wine triangle line). Here we set the Coulomb coupling strength λ = 0.95 *ω*_*m*_, the phase of the pump driving *θ* = 0, the temperature of environment *T* = 4 mK, and the laser driving power *P* = 50 mW.

We now show the effect of the gain of the OPA *C*_*g*_ on the entanglement between the oscillators. We fix the Coulomb coupling strength *λ* = 0.95 *ω*_*m*_, the phase of the pump driving *θ* = 0, the temperature of environment *T* = 4 mK, and the laser driving power *P* = 50 mW. The logarithmic negativity *E*_*N*_ as a function of the normalized detuning Δ/*ω*_*m*_ for six different values of *C*_*g*_ = 0 (red sphere line), *C*_*g*_ = 2 × 10^7^ Hz (blue triangle line), *C*_*g*_ = 5 × 10^7^ Hz (green circle line), *C*_*g*_ = 8 × 10^7^ Hz (magenta diamond line), *C*_*g*_ = 10 × 10^7^ Hz (olive pentagon line), and *C*_*g*_ = 12 × 10^7 ^Hz (wine triangle line) is shown in Fig. 3. From Fig. 3, we can find that the entanglement between the oscillators becomes more and more weaker and the entanglement region becomes more and more narrower with the increase of the gain of the OPA *C*_*g*_ compared with the case of in the absence of OPA (*C*_*g*_ = 0). This is because increasing the parametric gain *C*_*g*_ would lead to larger amount of squeezing of the cavity field when the phase of the pump driving is set to *θ* = 0^38^. As we all know, the radiation pressure exerted on the oscillator *B*_1_ by the cavity field depends on the number operator and then it is sensitive to the photon statistics of the intracavity field. The photon statistics can be calculated from the quantum Langevin Eq. (9). It can be proved that the Wigner function *W* of the intracavity field is Gaussian of the form $\exp [\mu {(\alpha -{c}_{s})}^{2}+\nu {({\alpha }^{\ast }-{c}_{s}^{\ast })}^{2}+\eta (\alpha -{c}_{s})({\alpha }^{\ast }-{c}_{s}^{\ast })]$, with *μ*, *v*, *η* determined by *κ*, Δ, *C*_*g*_, *θ*, etc. The photon number distribution associated with such a Gaussian Wigner function depends in an important way on the parameter *μ* and the inequality of *μ* and *v* ^64^ and the latter depend on *C*_*g*_ ≠ 0 or the presence of OPA in the cavity. The parametric squeezing processes inside the cavity change the quantum statistics of the field and larger amount of squeezing increases the photon number in the cavity, which lead to a stronger radiation pressure acting on the oscillator *B*_1_. However, besides the radiation pressure, there is another interaction, i.e., Coulomb coupling with the oscillator *B*_2_, acts on the oscillator *B*_1_ simultaneously. As to the oscillator *B*_1_, there exists the competing effect between the radiation pressure interaction and Coulomb interaction. So increasing the parametric gain *C*_*g*_ corresponds to a stronger radiation pressure, which seems to be equivalent to decrease the Coulomb coupling strength between the two separated oscillators in space in the absence of OPA (*C*_*g*_ = 0), as shown in Fig. 2(a). Additionally, the position of the maximal entanglement moves to right with the increase of gain *C*_*g*_ due to the fact that the injection of OPA strengths the steady intracavity field and in turn changes the effective deduning Δ. This is very similar to such the case of the weaker Coulomb coupling strength in the absence of OPA as shown in Fig. 2(a).

**Figure 4:**
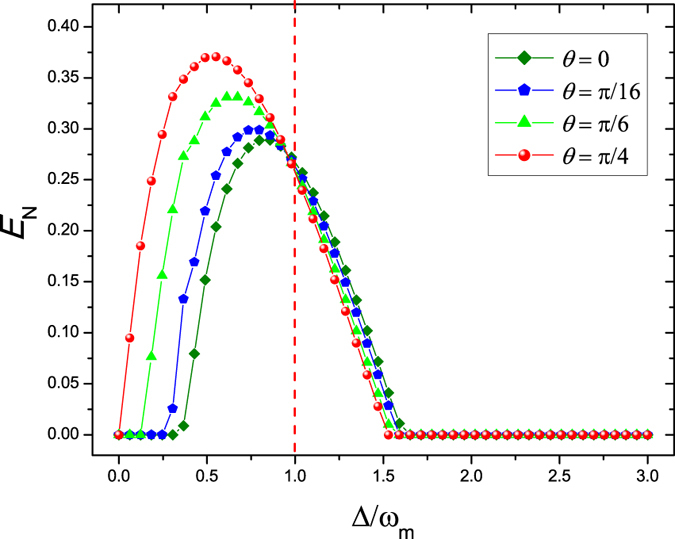
Plot of the logarithmic negativity *E*_*N*_ as a function of the normalized detuning Δ/*ω*_*m*_ for four different values of the phase of the pump driving the OPA *θ* = 0 (olive diamond line), *θ* = *π*/16 (blue pentagon line), *θ* = *π*/6 (green triangle line), and *θ* = *π*/4 (red sphere line). Here we set the Coulomb coupling strength *λ* = 0.95 *ω*_*m*_, the gain of the OPA *C*_*g*_ = 12 × 10^7^ Hz, the temperature of environment *T* = 4 mK, and the laser driving power *P* = 50 mW.

We next examine the effect of the phase of the pump driving the OPA *θ* on the entanglement between the oscillators. We fix the gain of the OPA *C*_*g*_ = 12 × 10^7^ Hz and other parameters are as same as the Fig. 3. The logarithmic negativity *E*_*N*_ as a function of the normalized detuning Δ/*ω*_*m*_ for four different values of the phase of the pump driving the OPA *θ* = 0 (olive diamond line), *θ* = *π*/16 (blue pentagon line), *θ* = *π*/6 (green triangle line), and *θ* = *π*/4 (red sphere line) is shown in Fig. 4. It can be clearly seen that the entanglement between the oscillators becomes more and more stronger with the increase of the phase *θ* when Δ < *ω*_*m*_ for the fixed gain *C*_*g*_ of the OPA. This is due to the fact that increasing the phase of the pump driving *θ* corresponds to a smaller amount of squeezing of the cavity field^38^ and the Coulomb interaction becomes the dominant factor compared with the radiation pressure interaction for the oscillator *B*_1_. So the influence of phase driving *θ* on the entanglement between the two separated oscillators in space is completely opposite compared with the function of the parametric gain *C*_*g*_ as shown in Fig. 3. We can also find that when Δ = *ω*_*m*_, all curves are intersected in one point. This can be interpreted as driving the system by the red-detuned laser Δ = *ω*_*m*_ in the resolved sideband limit makes the optomechanical interaction between the cavity field and the oscillator *B*_1_ like a beam-splitter interaction. In such case, the competing effect between the radiation pressure interaction and the Coulomb interaction acting on the oscillator *B*_1_ maintains a balance.

**Figure 5:**
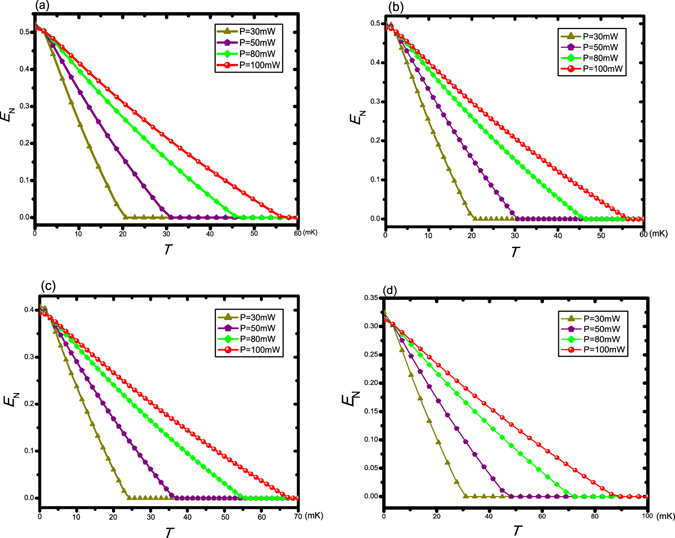
Plot of the logarithmic negativity *E*_*N*_ as a function of the temperature for four different values of the laser power *P* = 30 mW (dark yellow triangle line), *P* = 50 mW (purple pentagon line), *P* = 80 mW (green diamond line), and *P* = 100 mW (red sphere line). Here we set *λ* = 0.95 *ω*_*m*_, Δ = 0.75 *ω*_*m*_, *θ* = *π*/16, and *C*_*g*_ = 0, 2 × 10^7^ Hz, 8 × 10^7^ Hz, 12 × 10^7^ Hz, respectively, in (**a**–**d**).

Hence, resorting to the squeezing of the cavity field generated by an OPA inside the cavity, we successfully analyze the effect of squeezed light driving on this classical-to-quantum transition behavior instead of injecting the squeezed field directly. In the following, we show the effect of the Brownian noise on the entanglement between the oscillators, i.e., the effect of the temperature of the environment. The logarithmic negativity *E*_*N*_ as a function of the temperature for four different values of the laser power *P* = 30 mW (dark yellow triangle line), *P* = 50 mW (purple pentagon line), *P* = 80 mW (green diamond line), and *P* = 100 mW (red sphere line) when the Coulomb coupling strength *λ* = 0.95 *ω*_*m*_, Δ = 0.75 *ω*_*m*_, and the phase of the pump driving *θ* = *π*/16 is plotted in Fig. 5, where *C*_*g*_ is set as 0, 2 × 10^7^ Hz, 8 × 10^7^ Hz, and 12 × 10^7^ Hz, respectively. It can be concluded that for the fixed gain *C*_*g*_ of the OPA, as the temperature of the environment increases, the amount of entanglement monotonically decreases due to the environment thermal noise induced decoherence which is as expected. The higher the temperature of the environment is, the stronger the thermal noise is. Then the entanglement between two oscillators is submerged by the strong thermal noise. The critical temperature of the entanglement is improved with the increase of the laser driving power for the fixed gain *C*_*g*_ of the OPA and the numerical simulation results indicate that the robustness is obviously increased compared with previous schemes^18, 27^. While for the fixed laser driving power, the critical temperature of the entanglement is higher with respect to the larger gain *C*_*g*_ of the OPA. More importantly, from Fig. 5(a,d), it is remarkable that compared with the case of absence of the OPA, the presence of OPA obviously increases the robustness against the environment temperature of this classical-to-quantum transition behavior between the oscillators separated in space. It has also been verified that the OPA inside a cavity can considerably improve the cooling of the oscillator by radiation pressure^36^. Additionally, the relevant experimental investigation in such temperature requirement can be explored in the circuit cavity electromechanics^65^, which is easily cooled to the temperature below 100 mK.

**Figure 6:**
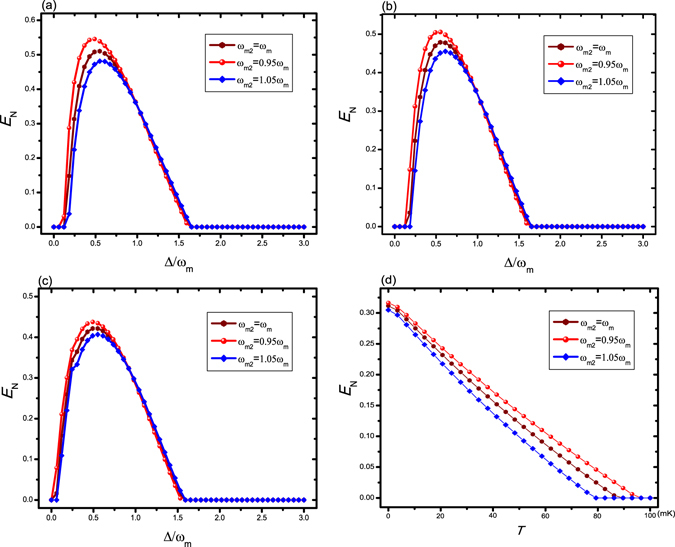
Plot of the logarithmic negativity *E*_*N*_ when the frequencies of two oscillators are different. (**a**) *λ* = 0.95 *ω*_*m*_ and other parameters are same with Fig. 2; (**b**) *C*_*g*_ = 2 × 10^7^ Hz and other parameters are same with Fig. 3; (**c**) *C*_*g*_ = 8 × 10^7^ Hz, *θ* = *π*/4, and other parameters are same with Fig. 4; (**d**) *P* = 100 mW and other parameters are same with Fig. 5(d).

In practice, it is hard to achieve two totally identical oscillators in experiment. So it is greatly necessary to consider the case in which the two separated oscillators in space have different frequencies (*ω*_*m*1_ ≠ *ω*_*m*2_). For this reason, we plot the logarithmic negativity *E*_*N*_ when the two oscillators have different frequencies in Fig. 6, where we set *ω*_*m*1_ = *ω*_*m*_, *ω*_*m*2_ = 0.95 *ω*_*m*_ (red sphere line), and *ω*_*m*2_ = 1.05 *ω*_*m*_ (blue diamond line), respectively. In order to make a comparison, we also plot *E*_*N*_ when *ω*_*m*1_ = *ω*_*m*2_ = *ω*_*m*_ (purple hexagram line). The figures show that whether the OPA exists or not, the influence of 5% frequency deviation of *ω*_*m*2_ on the entanglement between the two oscillators separated in space is similar. When the frequency of *B*_2_ *ω*_*m2*_ = 0.95 *ω*_*m*_ (red sphere line), the entanglement between the oscillators is slightly enhanced. While when *ω*_*m*2_ = 1.05 *ω*_*m*_ (blue diamond line), the entanglement is slightly decreased and optimal entanglement is slightly shifted towards higher detuning value. More importantly, the robustness of entanglement against environment temperature is stronger along with the enhanced entanglement when *ω*_*m*2_ = 0.95 *ω*_*m*_. The above discussion indicates that the slight frequency deviation between the oscillators not only cannot affect the classical-to-quantum transition behavior between two oscillators separated in space from experimental point, but also implies that we can control the strength of entanglement and the optimal entanglement occurrence through adjusting the frequencies of the two oscillators.

Last but not least, we note that Eq. (4) does’t contain the quadratic terms ${q}_{j}^{2}(j=1,2)$ which arise from the Eq. (2), i.e., the effect of the frequency shift caused by the Coulomb interaction between the two charged oscillators. In practice, the influence of the quadratic terms ${q}_{j}^{2}(j=1,2)$ in Eq. (2) is to readjust the frequency of the oscillators. Hence, it is significant necessary to discuss the effect of the frequency shift caused by the Coulomb interaction. During the above analysis, we have chosen the maximum coefficient of *ħq*_1_ *q*_2_ term as 0.95 *ω*_*m*_. To realize the present scheme, therefore, we should choose the frequency of the oscillator once again to supplement the negative frequency shift due to the Coulomb interaction. The new frequency of the oscillator approximately approaches to 280 *π*MHz because it is just the required oscillator in our scheme when taken the frequency shift due to the Coulomb interaction into account. It is worthy to point out that, with the fast development of the micro-nano manufacturing technology, the high-quality and high frequency micro-nano oscillator has been arisen in experiment. Specially, the oscillator of the order of magnitude of the gigahertz has been reported in experiment^66^.

In addition, the technology of generation of squeezed photons via the second-order nonlinearity in the cavity has been quite mature now. In 1986, Wu *et al*. has realized the squeezing of photons via the degenerate parametric amplifier in the cavity experimentally^67^. Methods for detection of entanglement have been discussed^17, 20^, and the entanglement properties between the oscillators can be verified by experimentally measuring the corresponding covariance matrix. It can be achieved by combining existing experimental techniques. The mechanical position and momentum can be measured with the setup proposed in ref. 49 in which via adjusting the detuning and bandwidth of an additional adjacent cavity, both position and momentum of the oscillator can be measured by homodyning the output of the second cavity.

## Conclusion

In conclusion, we have proposed a scheme to show that the system consisting of two macroscopic oscillators separated in space which are coupled through Coulomb interaction displays the classical-to-quantum transition behavior under the action of optomechanical coupling interaction. Our investigation indicates that once the optomechanical coupling interaction disappears, the entanglement between the two separated oscillators disappears accordingly and the system will return to classical world even though there exists sufficiently strong Coulomb coupling between the oscillators. Resorting to the squeezing of the cavity field generated by an OPA inside the cavity, we also consider the case of squeezed light driving and discuss the effect of squeezed light driving on this classical-to-quantum transition behavior instead of injecting the squeezed field directly. We find that even though the squeezed light driving cannot enhance the strength of the classical-to-quantum transition behavior, it considerably increases the robustness against the environment temperature of this transition behavior. We also find that in the case of ordinary light driving or squeezed light driving, the slight frequency deviation between the oscillators not only cannot affect the occurrence of this classical-to-quantum transition behavior, but also implies to control the strength of entanglement and optimal entanglement occurrence through adjusting the frequencies of the two separated oscillators. In current experimentally feasible regimes, the results of numerical simulation show that the present scheme is feasible and practical and has stronger robustness compared with previous schemes. It is promising that the scheme might possibly help us to further clarify and grasp the classical-quantum boundary.
